# The cyanobacterial nitrogen fixation paradox in natural waters

**DOI:** 10.12688/f1000research.10603.1

**Published:** 2017-03-09

**Authors:** Hans Paerl

**Affiliations:** 1 Institute of Marine Sciences, University of North Carolina at Chapel Hill, Morehead City, NC, USA

**Keywords:** cyanobacteria, nitrogen fixation, freshwater, marine

## Abstract

Nitrogen fixation, the enzymatic conversion of atmospheric N (N _2_) to ammonia (NH _3_), is a microbially mediated process by which “new” N is supplied to N-deficient water bodies. Certain bloom-forming cyanobacterial species are capable of conducting N _2 _fixation; hence, they are able to circumvent N limitation in these waters. However, this anaerobic process is highly sensitive to oxygen, and since cyanobacteria produce oxygen in photosynthesis, they are faced with a paradoxical situation, where one critically important (for supporting growth) biochemical process is inhibited by another.

N _2_-fixing cyanobacterial taxa have developed an array of biochemical, morphological, and ecological adaptations to minimize the “oxygen problem”; however, none of these allows N _2_ fixation to function at a high enough efficiency so that it can supply N needs at the ecosystem scale, where N losses via denitrification, burial, and advection often exceed the inputs of “new” N by N _2_ fixation. As a result, most marine and freshwater ecosystems exhibit chronic N limitation of primary production. Under conditions of perpetual N limitation, external inputs of N from human sources (agricultural, urban, and industrial) play a central role in determining ecosystem fertility and, in the case of N overenrichment, excessive primary production or eutrophication. This points to the importance of controlling external N inputs (in addition to traditional phosphorus controls) as a means of ensuring acceptable water quality and safe water supplies.

Nitrogen fixation, the enzymatic conversion of atmospheric N _2_ to ammonia (NH _3_) is a  microbially-mediated process by which “new” nitrogen is supplied to N-deficient water bodies.  Certain bloom-forming cyanobacterial species are capable of conducting N _2 _fixation; hence they are able to circumvent nitrogen limitation in these waters. However, this anaerobic process is highly sensitive to oxygen, and since cyanobacteria produce oxygen in photosynthesis, they are faced with a paradoxical situation, where one critically-important (for supporting growth) biochemical process is inhibited by another. Diazotrophic cyanobacterial taxa have developed an array of biochemical, morphological and ecological adaptations to minimize the “oxygen problem”; however, none of these allows N _2_ fixation to function at a high enough efficiency so that it can supply N needs at the ecosystem scale, where N losses via denitrification, burial and advection often exceed the inputs of “new” N by N _2_ fixation.

As a result, most marine and freshwater ecosystems exhibit chronic N-limitation of primary production.  Under conditions of perpetual N limitation, external inputs of N from human sources (agricultural, urban, industrial) play a central role in determining ecosystem fertility and in the case of N-overenrichment, excessive primary production, or eutrophication. This points to the importance of controlling external N inputs (in addition to traditional phosphorus controls) as a means of ensuring acceptable water quality and safe water supplies.

Nitrogen fixation, the biochemical conversion of “inert” atmospheric N (N _2_) to biologically available ammonia (NH _3_), is a microbially mediated process of global significance because it provides “new” N to aquatic ecosystems in which biological production is often controlled by N availability ^1, 2^. N _2_ fixation is an anaerobic process carried out by specific prokaryotes, including heterotrophic and chemolithotrophic bacteria and some cyanobacteria (blue-green algae) ^3^. The process likely evolved during the oxygen (O _2_)-devoid Precambrian period some 2+ billion years ago ^4, 5^. Of the N _2_-fixing microbial taxa, the cyanobacteria are of particular biogeochemical and ecological interest because they were also the first O _2_-evolving photosynthetic organisms on Earth ^6^; their proliferation during this period is thought to be an evolutionary “milestone” because it led to the generation of an O _2_-rich atmosphere, a prerequisite for the evolution of O _2_-requiring fungi, bacteria, animals, and higher plant species on our planet ^6^.

Ironically, the development of an O _2_-rich atmosphere, hydrosphere, and pedosphere constituted a formidable biochemical challenge for the cyanobacteria because, while they were capable of fixing N _2_, the process had to be confined to an O _2_-free micro-environment ^7^. This requirement posed a serious dilemma, especially for aquatic cyanobacteria, because they require illuminated conditions in surface waters, but the high ambient O _2_ levels produced by photosynthesis in these waters also represents an environmental barrier to O _2_-sensitive N _2_ fixation. Over their long evolutionary history, cyanobacteria have developed biochemical and structural adaptations as well as biotic associations in order to optimize N _2_ fixation while relying on oxygenic photosynthesis to provide energy and organic carbon (C) compounds to support metabolism and growth. The adaptions include (1) confining N _2_ fixation to night-time when photosynthesis is “turned off”, (2) forming colonies and aggregates to reduce illumination and form low-O _2_ “microzones”, (3) participating as endosymbionts in biological associations, and (4), forming heterocysts (non-photosynthetic, O _2_-free cells) in some filamentous taxa, which allows N _2_ fixation to proceed while receiving photo-reductant and organic C through photosynthesis from adjacent cells ^8^.

These are all remarkably clever adaptations to a modern-day oxic biosphere, which help circumvent the “O _2_ problem” ^6^. From an ecosystem perspective, they have allowed N _2_-fixing species to provide biologically available N from the vast reservoir of atmospheric N _2_. However, on the ecosystem scale, recent N budget analyses indicate that N _2_ fixation inputs fall far short of meeting ecosystem requirements when biologically available N inputs (from terrestrial and atmospheric sources) and losses (via denitrification, sedimentation and burial, and advection) are considered ^9– 11^. As a result, freshwater, estuarine, and marine systems are often chronically N deficient ^11– 17^. Pervasive N limitation has many implications for ecosystem function, especially when excessive external nutrient inputs lead to accelerating primary production (eutrophication), harmful algal blooms, and excessive O _2_ consumption (hypoxia). If chronic N-limited conditions prevail in water bodies and N _2_ fixation cannot meet ecosystem N requirements, then external N inputs often supply N to support eutrophication and its unwanted symptoms. From a management perspective, this means that the growing global glut of N inputs from agricultural, urban, and industrial sources ^14, 18– 20^ needs to be controlled, in addition to the broadly accepted phosphorus (P) input constraints, in order to protect our waterways and water supplies.

Why does N _2_ fixation fall short of meeting ecosystem demands? Apparently, this process does not operate at sufficient rates in a modern-day, oxic world to compensate for losses via burial, export, and denitrification, even though it is protected and optimized by the various biological adaptations mentioned above. It is counteracted at larger scales by biogeochemical processes, such as denitrification, that run in the opposite direction (NO _3_ → N _2_). The N _2_-fixing process is an energy-demanding one, requiring 16 ATP molecules to fix one molecule of N _2_^3^. In cyanobacteria, this energy demand has to be met by photosynthesis, while in non-photosynthetic bacteria, organic matter and redox reactions serve as energy sources ^3^. In highly productive (eutrophic), turbid waters where cyanobacteria and bacteria thrive, the availability of photosynthetically active radiation (PAR: 400–700 nm) is often restricted, causing a radiant energy deficit and suboptimal N _2_ fixation rates. Secondly, cyanobacteria taxa that dominate in eutrophic waters often accumulate as thick surface “blooms”, in part to circumvent light limitation in subsurface waters ^11^. High rates of photosynthesis in such blooms lead to O _2_ supersaturation, often in excess of 200% saturation ^21^. These ambient O _2_ levels inhibit N _2_ fixation *in situ*, even in heterocystous taxa ^22, 23^. Thirdly, N _2_ fixation requires high levels of P (to support the energetics, e.g. ATP formation and nucleic acid production) and metals, most prominently iron (Fe), which is a co-factor in the enzyme complex nitrogenase ^3^. In highly oxygenated surface waters, Fe occurs as the insoluble and biologically unavailable Fe ^3+^ ion that may lead to Fe-limited conditions ^24^. Lastly, wind-induced turbulence and vertical mixing can reduce N _2_ fixation potential by disrupting colonies and aggregates and enhancing inward diffusion of O _2_ ( Figure 1) ^25^ and deepening the mixed layer, reducing light availability.

**Figure 1.**
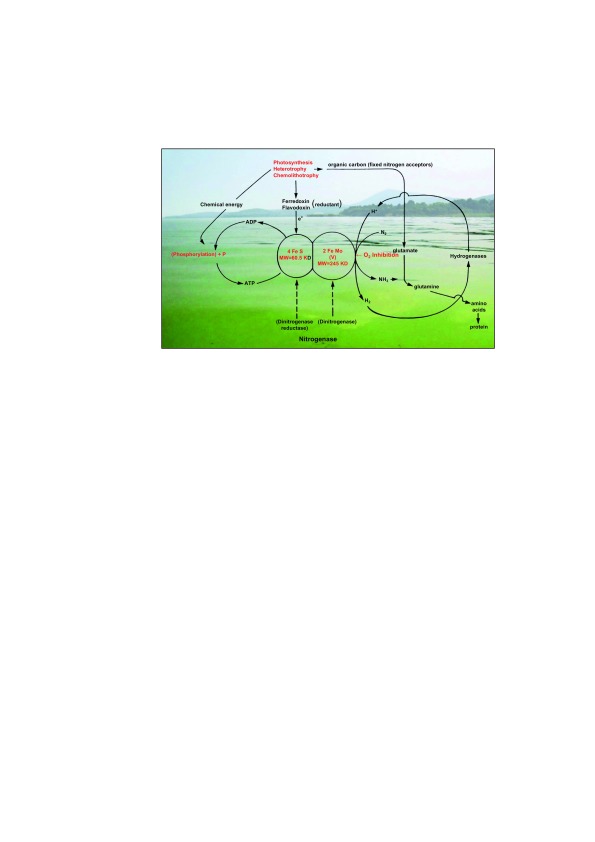
The nitrogen fixing process, as mediated by cyanobacteria (utilizing oxygenic photosynthesis as an energy and carbon source) as well as heterotrophic and chemolithotrophic microorganisms, in eutrophic surface waters. Potential environmental controls, including phosphorus (P) and iron (Fe) availability, energy sources, and dissolved oxygen inhibition, are shown in red. The background photo is of an O _2_-supersaturated (during daytime) cyanobacterial surface bloom in Lake Taihu, China. Photograph by H. Paerl.

Thus, while N _2_ fixation converts inert N _2_ into biologically available NH _3_ to support aquatic fertility in a remarkable fashion, it faces multiple constraints and limitations in aquatic environments, especially in surface waters, which are often N limited. Geochemists, some limnologists, and a few oceanographers have assumed that as long as P and Fe are readily available, N _2_ fixation should make up for an N deficit, given the unlimited supply of N _2_ available ^26, 27^. However, this assumed linear stoichiometric relationship is not straightforward. Major environmental factors constrain this process, preventing it from functioning at optimal rates and supplying complete ecosystem N requirements ^8, 11^. As a result, much of the world’s marine and freshwater environments remain chronically N deficient. In practical (management) terms, this limitation means that external inputs of N play a key role in providing adequate and excessive fertility (eutrophication) of many freshwater and most marine ecosystems ^11, 15, 16^. Tremendous increases in anthropogenically generated bioavailable N in the form of synthetic (Haber process) fertilizers, agricultural, industrial, and urban wastes, and N _2_ emissions (as both oxides and reduced forms of N) far overshadow biological fixation of N _2_ in providing available N to receiving waters. Effective future management and protection of our fresh and marine waters will depend on the control of external inputs of both N and P ^11, 27^ instead of depending on the more traditional approach of controlling P inputs without N restrictions ^28^.
